# A novel way to secure a chest drain

**DOI:** 10.1308/rcsann.2014.96.1.82

**Published:** 2014-01

**Authors:** D Maritz, C McLauchlan

**Affiliations:** Royal Devon and Exeter NHS Foundation Trust,UK

## BACKGROUND

Dislodgement of chest drains has the potential to cause serious morbidity and even mortality. Many techniques for securing chest drains to the skin have been described.^1–4^ We descibe a simple yet effective technique for securing large bore drains to the skin using a single suture.

## TECHNIQUE

**Figure 1:**
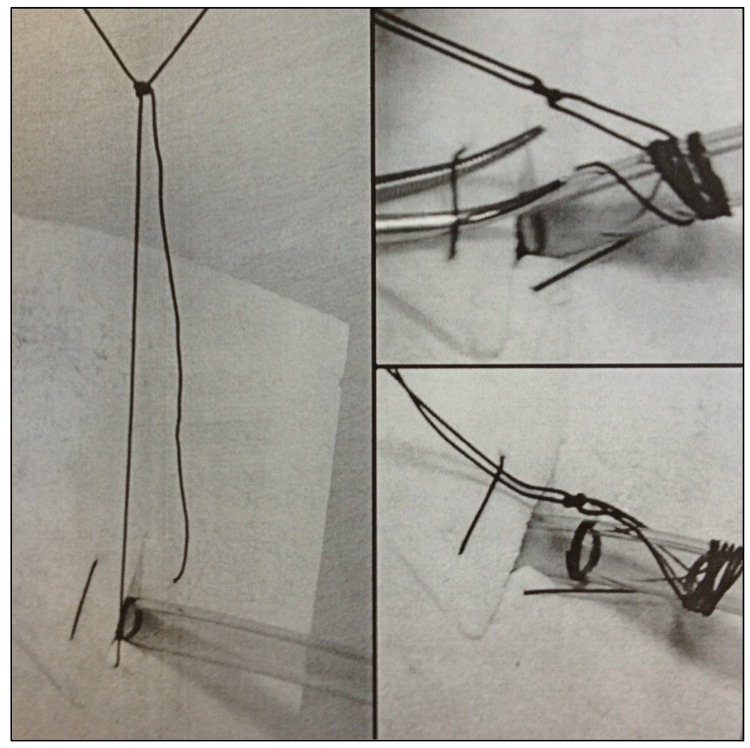
Securing the drain

After drain insertion, a horizontal mattress suture is placed. A simple knot is placed distally half way along the two free ends. The two threads are wrapped tightly together the same way around the tube up to the level of the knot. The free ends of the knot are then passed proximally under the loop of the skin suture (Fig 1).

**Figure 2:**
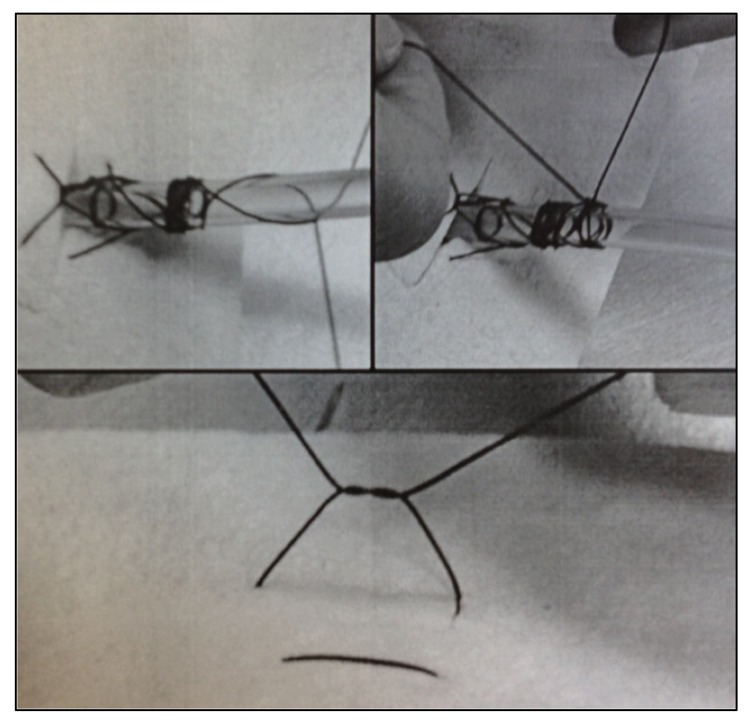
Securing the drain and closing the skin

The suture is pulled firmly, and the free ends are separated and wrapped around the tube in opposite directions a few times. The two ends are tied together tightly using a surgeon’s knot. Traction on the tube tightens the wound and the grip on the tube (Fig 2). The same suture is used to close the skin by unwrappping the threads and cutting them just proximal to the first knot placed (Fig 2).

## DISCUSSION

The British Thoracic Soceity guidelines recommend the use of two sutures: one to secure the tube and the other to close the skin.^5^ Complicated purse string methods should be avoided as they can cause unsightly scars.
